# Institutionalized data quality assessments: *a critical pathway to improving the accuracy of integrated disease surveillance data in Sierra Leone*

**DOI:** 10.1186/s12913-020-05591-x

**Published:** 2020-08-07

**Authors:** Charles Njuguna, Mohamed Vandi, Malimbo Mugagga, Joseph Kanu, Evans Liyosi, Alexander Chimbaru, Wilson Gachari, Victor Caulker, Stephen Sesay, Jane Githuku, Zabulon Yoti, Ali Ahmed Yahaya, Ambrose Talisuna, Ibrahima Socé Fall

**Affiliations:** 1 World Health Organization Sierra Leone, Freetown, Sierra Leone; 2 Ministry of Health and Sanitation Sierra Leone, Freetown, Sierra Leone; 3 World Health Organization Regional Office for Africa, Brazzaville, Congo

**Keywords:** Public health surveillance, Data quality, Assessment, Sierra Leone

## Abstract

**Background:**

Public health agencies require valid, timely and complete health information for early detection of outbreaks. Towards the end of the Ebola Virus Disease (EVD) outbreak in 2015, the Ministry of Health and Sanitation (MoHS), Sierra Leone revitalized the Integrated Disease Surveillance and Response System (IDSR). Data quality assessments were conducted to monitor accuracy of IDSR data.

**Methods:**

Starting 2016, data quality assessments (DQA) were conducted in randomly selected health facilities. Structured electronic checklist was used to interview district health management teams (DHMT) and health facility staff. We used malaria data, to assess data accuracy, as malaria was endemic in Sierra Leone. Verification factors (VF) calculated as the ratio of confirmed malaria cases recorded in health facility registers to the number of malaria cases in the national health information database, were used to assess data accuracy. Allowing a 5% margin of error, VF < 95% were considered over reporting while VF > 105 was underreporting. Differences in the proportion of accurate reports at baseline and subsequent assessments were compared using Z-test for two proportions.

**Results:**

Between 2016 and 2018, four DQA were conducted in 444 health facilities where 1729 IDSR reports were reviewed. Registers and IDSR technical guidelines were available in health facilities and health care workers were conversant with reporting requirements. Overall data accuracy improved from over- reporting of 4.7% (VF 95.3%) in 2016 to under-reporting of 0.2% (VF 100.2%) in 2018. Compared to 2016, proportion of accurate IDSR reports increased by 14.8% (95% CI 7.2, 22.3%) in May 2017 and 19.5% (95% CI 12.5–26.5%) by 2018. Over reporting was more common in private clinics and not- for profit facilities while under-reporting was more common in lower level government health facilities. Leading reasons for data discrepancies included counting errors in 358 (80.6%) health facilities and missing source documents in 47 (10.6%) health facilities.

**Conclusion:**

This is the first attempt to institutionalize routine monitoring of IDSR data quality in Sierra Leone. Regular data quality assessments may have contributed to improved data accuracy over time. Data compilation errors accounted for most discrepancies and should be minimized to improve accuracy of IDSR data.

## Background

Public health surveillance data is used to monitor disease trends, detect outbreaks and trigger response activities. It also guides allocation of resources, evaluation of public health interventions, policies and strategies [1]. Policy makers and public health agencies rely on good quality data to make decisions. In infectious disease surveillance and response, reliable, valid, timely, complete and accurate health information is essential for early detection and control of outbreaks [2]. The revised International Health Regulations (2005), require state parties to build public health capacity to detect, report, and respond to public health threats [3, 4]. This can only be achieved through robust public health surveillance systems that generate high quality data. Emphasis on improved accountability of donor funding in the health sector has also driven the need for accurate data to track progress of key indicators [5]. Demand for high quality data has led to an increase in the number of data quality assessments worldwide [6].

Data quality refers to those features and characteristics that ensure data are accurate and complete and that they convey the intended meaning. Data quality is measured both directly and indirectly [1]. Simple checks on the number of empty variables in medical records provide an idea of how complete and accurate the data are. Accuracy is measured by comparing summary reports to recounted values verified through formal data quality assessments [1, 7–9]. The validity of laboratory tests, training of persons who record information in the surveillance system, frequency of supervision and data management practices are indirect measures of data quality [1]. More comprehensive definitions of data quality have been described, such as breaking down data quality into dimensions of data, data use and data collection process. Each dimension is then assessed using attributes [6].

Good data quality remains a challenge especially in low resource settings. Assessments of implementation of IDSR in African countries have identified data quality issues such as late reporting [10], incomplete reporting [11] and data inconsistencies across reporting levels [12]. However, repeated assessments, feedback, data management trainings appear to improve data quality over time.

Sierra Leone had the highest number of cases during the Ebola outbreak in West Africa, 2014–2016 [13]. The outbreak led to a breakdown of the public health system including surveillance. Efforts to revitalize surveillance started with a rapid assessment of IDSR capacity in 2015. The assessment identified potential threats to data quality such as low reporting rates, unavailability of data collection and reporting tools and difficulties in data transmission. Starting January 2015, the Ministry of Health and Sanitation, with technical support from WHO and other partners, supported the country to restructure and operationalize the IDSR system. Technical guidelines were adapted and aligned to Africa regional IDSR guidelines (2010) and the international health regulations (2005). Also, health care workers were trained, data collection tools distributed, and necessary infrastructure provided [14]. At first, the IDSR system was paper based, whereby health facility IDSR focal persons would send weekly IDSR reports to the district health office through hand delivery, phone calls or short text messages. Officials in the district health office would then collate reports in an MS excel database and forward to national surveillance officials at the national level. The migration to electronic IDSR data transmission (e-IDSR) started in July 2016 at the district level, whereby weekly IDSR data received from health facilities were entered into the District Health Information Software (DHIS2) platform, and were thereafter accessible to officials at the national level including partners.

Starting May 2017, electronic reporting was piloted in health facilities in one district and by June 2019, IDSR reporting was done electronically in all health facilities. One year after the revitalized IDSR system was operationalized, we undertook periodic DQA of data generated through the IDSR system. We also explored reasons for discrepancies in recounted data and data available in DHIS2. Data on positive malaria cases were used for the exercise as malaria is endemic in all districts of Sierra Leone [15].

## Methods

### Design and study setting

Four retrospective assessments were conducted in selected health facilities in all districts of Sierra Leone. IDSR data collected in July 2016, May 2017, November 2017 and October 2018 was reviewed. While the MoHS aimed to conduct two assessments per year, this was only achieved in 2017 due to time and personnel constraints. Thus, in 2016 and 2018, only one assessment was conducted per year. Health facilities were selected based on the health service level, whereby at least one hospital, two Community Health Centers (CHC), two Community Health Posts (CHP), and two Maternal Child Health Posts (MCHP) were included per district. The number of facilities increased in the third and fourth assessments. The DHMT would list all health facilities included in the IDSR reporting system, stratifying them by service level and ownership. For each type of facility, selection would be done using randomly generated computer numbers.

### Interviews and data validation

An electronic checklist was developed and uploaded onto tablets using the Open Data Kit (ODK) platform. In preparation for the visit, the office of the directorate of prevention and control (DPC) would send a notification of the DQA exercise to the district health management team (DHMT). This was to allow the team prepare a list of health facilities and avail two DHMT officials to join the national team. On the first day of the assessment, the national team would meet the members of the DHMT, give an overview of the DQA process and interview key informants in the DHMT. The team would record the number of positive malaria cases for each of the selected health facilities, as recorded in the district database. During the first assessment, IDSR data was stored in an MS Excel database at the district health office and DPC. From January 2017, the country shifted to electronic data transmission from district level whereby all IDSR data was uploaded directly to DHIS2 platform. During the second, third and fourth assessments the team would log onto the DHIS2 platform and retrieve the information for each selected health facility.

### Variables

The checklist was organized into seven sections. The first section was on availability and consistent use of registers at health facilities. Section two assessed the processes and tools that facilitated reporting in the IDSR. This included observations on availability of reporting tools, IDSR weekly reports and questions on the compiling of IDSR reports. The third section was on data analysis and interpretation and the fourth section had questions on feedback mechanisms used by the DHMTs. The remaining sections were on data validation, logistics management and reasons for discrepancies in data.

### Statistical analysis

Malaria positive cases reported over epidemiologic weeks 27–30 in 2016, 18–21 in 2017, 44–47 in 2017 and 40–43 in 2018 were used in the exercise. These periods corresponded to the four completed epidemiologic weeks prior to the DQA exercise. For each of the four weeks, the number of cases recorded in the health facility registers were counted and recorded in the assessment tool. Records of confirmed malaria cases in outpatient registers were used in facilities without laboratories. In hospitals with laboratories, malaria positive tests as recorded in the laboratory register were used. If rapid diagnostic tests were used in the outpatient department to test malaria, the data would be added to that recorded in the laboratory register. Next, the assessment team obtained the four weekly reports from the health facility and abstracted the number of confirmed malaria cases recorded. The last step involved abstracting the number of confirmed malaria cases for each health facility as recorded in the MS Excel database in district office or from the DHIS website.

### Calculation of accuracy

Data accuracy was determined by calculating a verification factor (VF) that was the ratio of the recounted (verified) value of positive malaria cases recorded in the health facility registers divided by value of positive malaria cases in the district office database in 2016 or DHIS database in 2017 and 2018.

$$ \mathrm{Verification}\ \mathrm{Factor}=\frac{\mathrm{Number}\ \mathrm{of}\ \mathrm{Malaria}\ \mathrm{Cases}\ \mathrm{Recounted}\ \mathrm{from}\ \mathrm{HF}\ \mathrm{Register}}{\mathrm{Number}\ \mathrm{of}\ \mathrm{Malaria}\ \mathrm{Cases}\ \mathrm{Recorded}\ \mathrm{in}\ \mathrm{DHIS}2}\times 100\% $$

Verification factors (VF) were calculated to compare concurrency between the verified counts of total malaria cases for each epidemiologic week in the registers (denoted as "a") with the number recorded in the health facility reports and the district database or DHIS (denoted as "b"). We adopted this method from the World Health Organization (WHO) guidelines for data quality assessment [7] VF=a/b*100.

Verification factor (VF) of > 100% was interpreted as underreporting while a verification factor of < 100% indicated over reporting. A 5% margin of error was considered acceptable (95 to 105%). We examined the difference in proportion of accurate reports in the first and fourth assessments using the Z-test for two proportions.

## Results

Four data quality assessments were conducted in 444 health facilities of which 390 (87.8%) were lower level facilities (CHC, CHP, MCHP) (Table 1), 47 (10.6%) were secondary and two (0.4%) were tertiary level hospitals. The number of health facilities included in the assessment increased from 79 in 2016 to 138 in 2018, thus improving the representativeness of the data. We reviewed 1729 weekly IDSR reports that is, 299 in the first assessment, 377 in the second assessment, 501 in the third assessment and 552 in the fourth assessment.

**Table 1:** Type of Health Facilities included in the data quality assessment, Sierra Leone, 2016–2018

Facility type	July 2016	May 2017	November 2017	October 2018	Total (%)
Community Health Centre (CHC)	27	28	40	41	136 (30.6)
Community health post (CHP)	23	27	40	44	134 (30.2)
Maternal and child health post (MCHP)	24	26	36	34	120 (27.0)
Hospital	5	14	14	16	49 (11)
Others (including clinics and not for profit organizations)	0	2	0	3	5 (1.1)
**Total**	**79**	**97**	**130**	**138**	**444 (100)**

### Data collection, collation, analysis and reporting

Although patient registers and IDSR reporting tools were available in most lower level health facilities they were less available in hospitals and laboratories. Hospital outpatient registers were used in 42 (85.7%) out of 49 hospitals. Registers used to record data for children aged < 5 years were used in 383 (98.2%) lower level health facilities while general clinic registers were used in 384 (98.5%) health facilities. Out of 144 health facilities with laboratories, 109 (75.7%) used laboratory registers to record patient information (Table 2). IDSR weekly reporting forms were available in 130 (94.2%), case based forms in 116 (84.1%) and technical IDSR guidelines in 122 (88.4%) health facilities (Table 3). There was a high level of awareness of IDSR reporting requirements as 401 (90.3%) of respondents correctly defined an epidemiologic week and 418 (94.1%) defined zero reporting correctly. Regular data analysis was conducted in 242 (54.5%) of the health facilities.

**Table 2:** Availability of standard inpatient and outpatient registers in health facilities, Sierra Leone, 2016–2018

Type of register	Recommended use/Description	No. of facilities required to use register	No. (%) of health facilities where register was available and in use
Hospital outpatient register	Used in hospitals to record information for all outpatient visits	49	42 (85.7)
Hospital in-patient register	Used in hospitals to record information on all admissions	49	40 (81.6)
Under 5 years register	Used in CHC, CHP and MCHP to record information on outpatient visits for children aged less than 5 years	390	383 (98.2)
General clinic register	Used in CHC, CHP and MCHP to record information on outpatient visits for persons aged > 5 years	390	384 (98.5)
Mother and neonate register	Used in all health facilities to record information on antenatal visits and child immunization	444	364 (82)
Maternity and delivery register	Used in all health facilities to record information on deliveries, maternal deaths	444	396 (89.2)
Laboratory register	Used only in health facilities (some CHCs and all hospitals) that have laboratories, to record information for all laboratory tests conducted in the laboratory, including malaria diagnostic tests	144	109 (75.7)

**Table 3:** Availability of IDSR technical guidelines and reporting tools, Sierra Leone, 2016–2018

	July 2016	May 2017	November 2017	October 2018
**IDSR tool**	**Frequency (%)** ***N*** **= 79**	**Frequency (%)** ***N*** **= 97**	**Frequency (%)** ***N*** **= 130**	**Frequency (%)** ***N*** **= 138**
Case based form	67 (84.8)	77 (79.3)	121 (93.1)	116 (84.1)
Line listing form	65 (82.3)	77 (79.3)	110 (84.6)	105 (76.1)
Weekly reporting form	75 (94.9)	94 (96.9)	124 (95.4)	130 (94.2)
Rumor log	41 (51.9)	54 (55.7)	84 (64.6)	90 (65.2)
Technical IDSR Guidelines	71 (89.9)	90 (92.8)	113 (86.9)	122 (88.4)

### Accuracy of data on malaria cases reported through the IDSR system by level of health care facility

The proportion of accurate reports was similar across all health facility types and was highest (52.5%) for data received from CHPs. Out of 49 reports received from clinics and health facilities owned by not-for-profit organizations 19 (38.8%) were over-reported. Under-reporting was more common in reports from CHPs (29%) and MCHPs (28.5%) (Table 4).

**Table 4:** Comparing Accuracy of IDSR weekly reports among Different Types of Health Facilities in Sierra Leone

Type of Health Facility	No. of health facilities	No. (%) of health facilities with Accurate Reports VF 95–105%	No. (%) of health facilities with over-reporting VF < 95%	No. (%) of health facilities with under- reporting VF > 105%
Community Health Center	528	276 (52.3)	129 (24.4)	123 (23.3)
Community Health Post	507	266 (52.5)	94 (18.5)	147 (29)
Maternal, Child Health Post	461	226 (49)	102 (22.1	133 (28.5)
Hospital	184	86 (46.7)	57 (31)	41 (22.3)
Clinics and not for profit organizations	49	24 (49)	19 (38.8)	6 (12.4)

### Improvements in data accuracy with repeated assessments

**Fig. 1:**
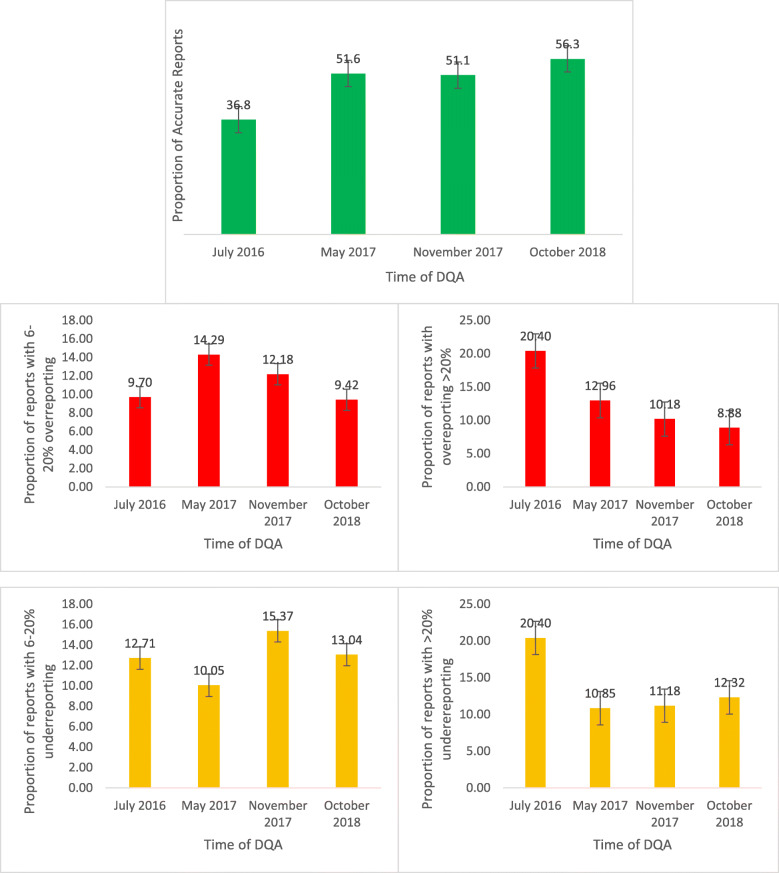
Comparing Accuracy of IDR weekly Reports Over time, Sierra Leone, 2016–2018

Overall data accuracy improved from over-reporting of 4.7% (VF of 95.3%) in the first assessment to under-reporting of 0.2% (VF of 100.2%) in the fourth assessment (Table 4). There was a significant improvement in the proportion of weekly reports with accurate malaria data from 36.8% in the first assessment to 56.3% in the fourth assessment (95% CI, 12.5, 26.5%) (Fig. 1). A significant improvement was observed between the first and second assessments [difference = 14.8% (95% CI 7.2, 22.3%)]. No significant change was observed between the second and third assessment [difference = 0.5% (95% CI -6.2, 7.2)] and between the third and fourth assessment [difference = 5.2% (95% CI -0.8, 11.2%)]. The number of districts with accurate data increased from six (46.2%) out of thirteen in the first assessment, eight (57.1%) in the second assessment, 10 (71.4%) in the third assessment and nine (64.3%) in the fourth assessment.

The largest discrepancies between recounted register data and DHIS data were 40.6% over reporting in Pujehun district in the first assessment and 25.7% underreporting in Tonkolili district in the fourth assessment (Table 5). The proportion of reports with over-reporting > 20% reduced from 20.4% in 2016 to 8.9% in 2018. Reports with underreporting > 20% also reduced from 20.4 to 12.3% in 2018 (Fig. 1). Weekly reports from Kailahun district were accurate during all assessments. Over reporting was more common in the first assessment (5/13 districts) and second assessments (4/14 districts) compared to the fourth assessment where underreporting was more common (3/14 districts) (Table 5).

**Table 5:** Comparing Accuracy of Malaria Data by district and time, Sierra Leone, 2016–2018

	July 2016			May 2017			November 2017			October 2018		
District	No. of malaria cases recounted HF register	No. of malaria cases recorded in District database /DHIS	VF Recounted data in HF register/ District database	No. of malaria cases recounted HF register	No. of malaria cases recorded in District database /DHIS	VF Recounted data in HF register/ District database	No. of malaria cases recounted HF register	No. of malaria cases recorded in District database /DHIS	VF Recounted data in HF register/ District database	No. of malaria cases recounted HF register	No. of malaria cases recorded in District database /DHIS	VF Recounted data in HF register/ District database
Bo	661	528	**125.2**	918	1027	**89.4**	1143	1158	98.7	1598	1569	101.8
Bombali	849	860	98.7	1088	1315	**82.7**	1134	1174	96.6	1785	1558	**114.6**
Bonthe	538	705	**76.3**	657	675	97.3	781	751	104.0	1026	986	104.1
Kailahun	1047	1033	101.4	1235	1235	100.0	749	780	96.0	943	972	97.0
Kambia	868	847	102.5	636	580	**109.7**	887	900	98.6	1469	1456	100.9
Kenema	932	898	103.8	699	711	98.3	1100	1003	**109.7**	1020	1021	99.9
Koinadugu	987	1092	**90.4**	854	868	98.4	1189	1210	98.3	1472	1349	**109.1**
Kono	1092	1221	**89.4**	1218	1153	**105.6**	986	1072	**92.0**	2380	2409	98.8
Moyamba	740	788	**93.9**	876	865	101.3	656	636	103.1	705	719	98.1
Port Loko	1023	1005	101.8	855	992	**86.2**	1243	1212	102.6	1966	1827	**107.6**
Pujehun	646	1087	**59.4**	764	746	102.4	588	663	**88.7**	986	941	104.8
Tonkolili	778	775	100.4	1223	1220	100.2	1199	1094	**109.6**	1357	1827	**74.3**
Western Area rural	–	–	**–**	731	778	**93.9**	1487	1476	100.7	1635	1565	104.5
Western Area Urban	996	863	**115.4**^**a**^	1116	1106	100.9	1865	1861	100.2	1326	1437	**92.3**
**Total**	**11,157**	**11,702**	**95.3**	**12,870**	**13,271**	**97**	**15,007**	**14,990**	**100.1**	**19,668**	**19,636**	**100.2**

^a^In 2016, Western Area was one district. In 2017, Western Area Rural District was formed

### Reasons for data discrepancies

Data compilation errors, made when generating monthly summaries of malaria cases, were the most common cause of discrepancies between recounted data and data in DHIS2. This was observed in 358 (80.6%) of all the health facilities and remained constant during all assessments. Other reasons for discrepancies were missing registers in 47 (10.6%) health facilities, failure to submit health facility IDSR weekly reports to the district office in 25 (5.6%) and failure to enter data into the DHIS2 database in 24 (5.4% health facilities (Table 6).

**Table 6:** Reasons for discrepancies in recounted data and data available in district database/ DHIS

Data Quality Error	Number (%) of health facilities where error was identified				
	July 2016 ***N*** = 79	May 2017 ***N*** = 97	November 2017 ***N*** = 130	October 2018 ***N*** = 138	Total ***N*** = 444
Counting Errors	58 (73.4)	70 (72.2)	111 (85.4)	119 (86.2)	358 (80.6)
Missing Source Documents (Registers)	7 (8.9)	13 (13.4)	14 (10.8)	13 (9.4)	47 (10.6)
Reports not submitted to the district	7 (8.9)	2 (2.1)	10 (7.7)	6 (4.3)	25 (5.6)
Health facility reports not entered into district database	8 (10.1)	0 (0)	9 (6.9)	7 (5.1)	24 (5.4)

## Discussion

This is among the first attempts to institutionalize routine monitoring of public health surveillance data quality in Sierra Leone and in the African region. By the fourth assessment, more than half of the weekly IDSR reports were within allowable accuracy limits and there was a reduction in the magnitude of the discrepancies between recounted data and data in the DHIS2.

A higher improvement in data accuracy was observed after the first assessment and stagnated thereafter. This is possibly due to the extensive dissemination of the DQA findings undertaken by the MoHS after the first assessment. Moreover, the first assessment was done during the post Ebola recovery period that was characterized by an increased focus on public health systems, and availability of technical and financial support for public health programs.

Improvement in data accuracy observed from these assessments implies that the regular data quality assessments may have contributed positively to data accuracy and is consistent with findings from repeated data quality audits conducted elsewhere [16–18]. Other contributors to improved data accuracy could be regular supportive supervision and shift to electronic reporting that were introduced simultaneously with the DQA. Availability of registers to record patient information in standard manner may also have had a positive impact on data accuracy. If patient data is captured in different tools with different formats, then aggregation may be erroneous. In addition, if registers are not available, patient information may not be recorded at all or may be recorded in informal registers and not transferred to the new register when it is available.

Difficulties in generating quality public health data in Africa are well documented. In Malawi, an undercount of 5.4% of the number of patients receiving antiretroviral treatment was found [9] which is within the range found in our study. More extreme deviations of 75.2% have been reported in Prevention of Mother to Child infection of HIV program sites in South Africa [8].

In our assessment, further analysis of health facility data revealed discrepancies in data that could have been masked if only the overall accuracy was considered. Disaggregation of data enabled us to calculate accuracy for each district and also check if accuracy varied by health facility type. We found that accuracy levels in several districts improved over time, probably as a result of feedback given to DHMTs during quarterly IDSR review meetings. Constant feedback on performance is a common approach used in many data quality improvement interventions and is part of the quality improvement process [17, 19]. While the type of health facility did not appear to influence data accuracy, we did observe that underreporting was more common in lower level peripheral health units and over reporting was more common in private clinics and not for profit owned facilities. Possible reasons for over-reporting of malaria cases may be the need to account for malaria commodities [20], duplication, or poor record keeping that leads to estimation of the actual cases when reporting.

Errors made by health care workers when counting and compiling reports were observed in all assessments and were the most important reason for discrepancies in data uploaded onto the DHIS2. Starting 2016, the MoHS, Sierra Leone, with support from WHO and other partners, undertook phased migration from paper based reporting to electronic reporting of IDSR data up to the health facility level. Currently, health facility IDSR data is uploaded directly onto DHIS2 platform, thus reducing discrepancies arising from transmission of reports to the district health office. Improvements in IDSR data quality will be dependent on the thoroughness of health care workers who compile and upload IDSR reports.

Our assessment had a few limitations. First, most of the health facilities included in the assessments were government owned health facilities that benefited from capacity building on IDSR and also from routine supervision. Data quality in such health facilities may be better compared to data quality in private health facilities. We are not able to examine possible differences in these two types of facilities owing to the small number of private health facilities included in this assessment. Secondly, the assessment focused on one attribute of data quality and cannot be used to assess validity of the data, as this requires more rigorous techniques. Although data on three epidemic prone diseases, namely measles, dysentery and acute flaccid paralysis were included in the assessments, the proportion of reports with data on the three conditions was too small to draw valid inference on the quality of data for these diseases.

## Conclusion

These assessments are the first attempt to institutionalize monitoring of IDSR data quality in Sierra Leone post EVD outbreak. Regular DQA contributed to gradual improvements in data accuracy and also enabled us identify data quality issues that need to be addressed. The shift to electronic surveillance system is likely to reduce transcription errors, thus focus should be on proper documentation, storage of patient information and accurate compilation of data at the health facility level. The MoHS should build the capacity of district health management team members to conduct DQA using the electronic checklist as this is more sustainable and can increase coverage to all health facilities. Future assessments should focus on the accuracy of data on epidemic prone diseases, private health facilities and more rigorous assessments of data validity.

## Data Availability

The data that support the findings of this study are available from the corresponding author upon reasonable request.

## Abbreviations

CHC: Child Health Center
CHP: Child Health Post
DHIS: District Health Information Software
DHMT: District Health Management Team
DQA: Data Quality Assessment
EVD: Ebola Virus Disease
IDSR: Integrated Disease Surveillance and Response
MCHP: Maternal Child Health Post
MoHS: Ministry of Health and Sanitation
ODK: Open Data Kit
VF: Verification Factor
WHO: World Health Organization
